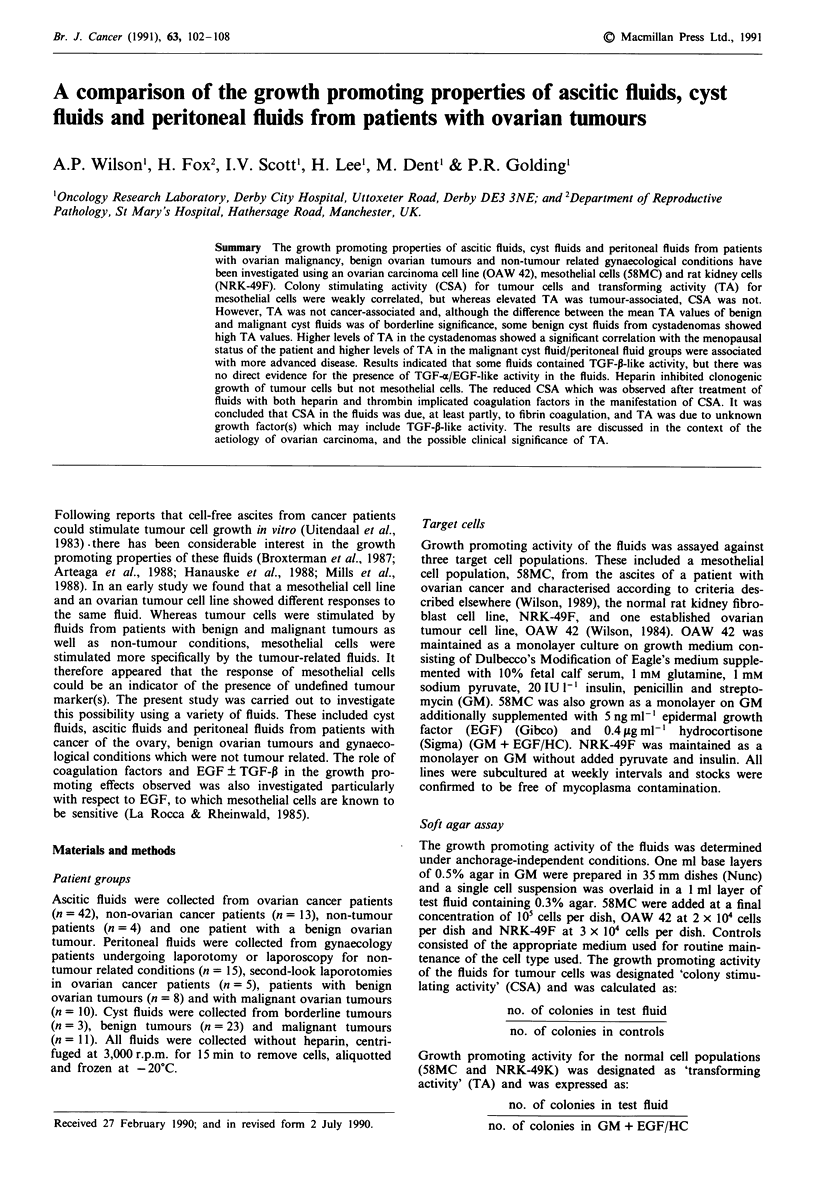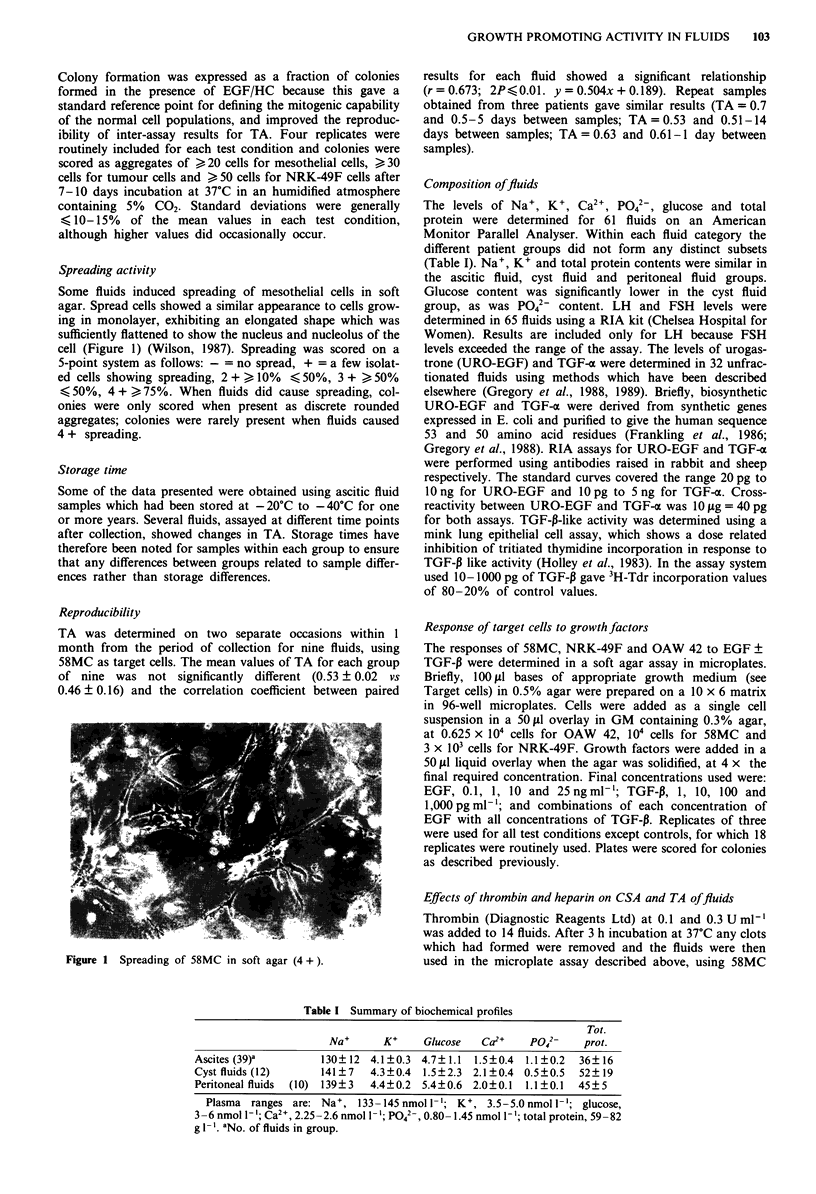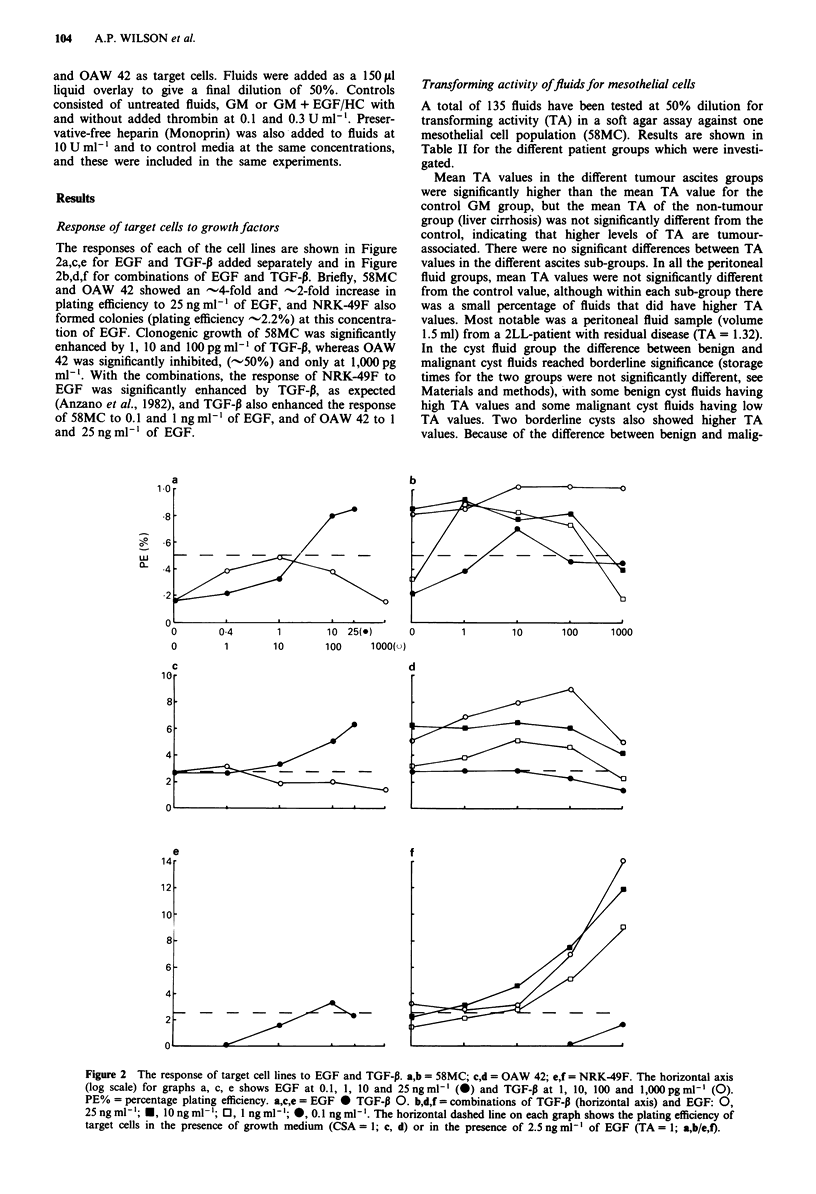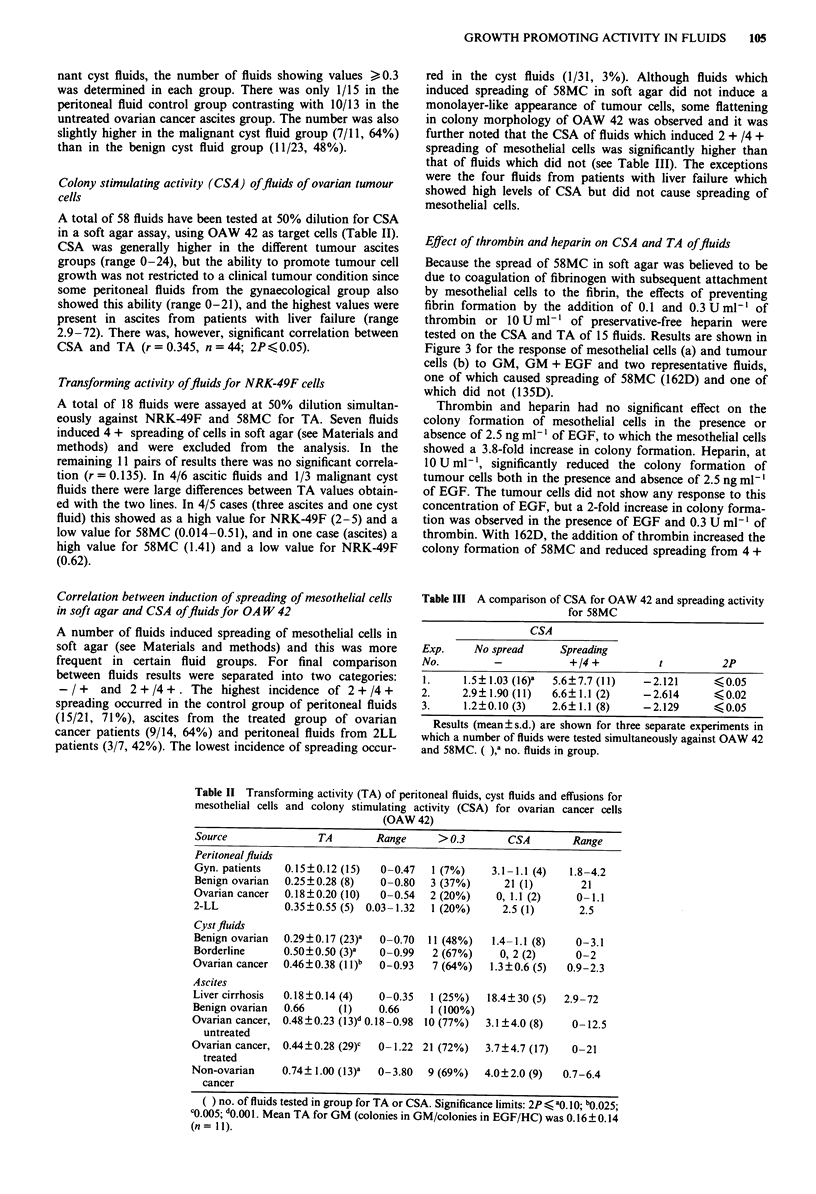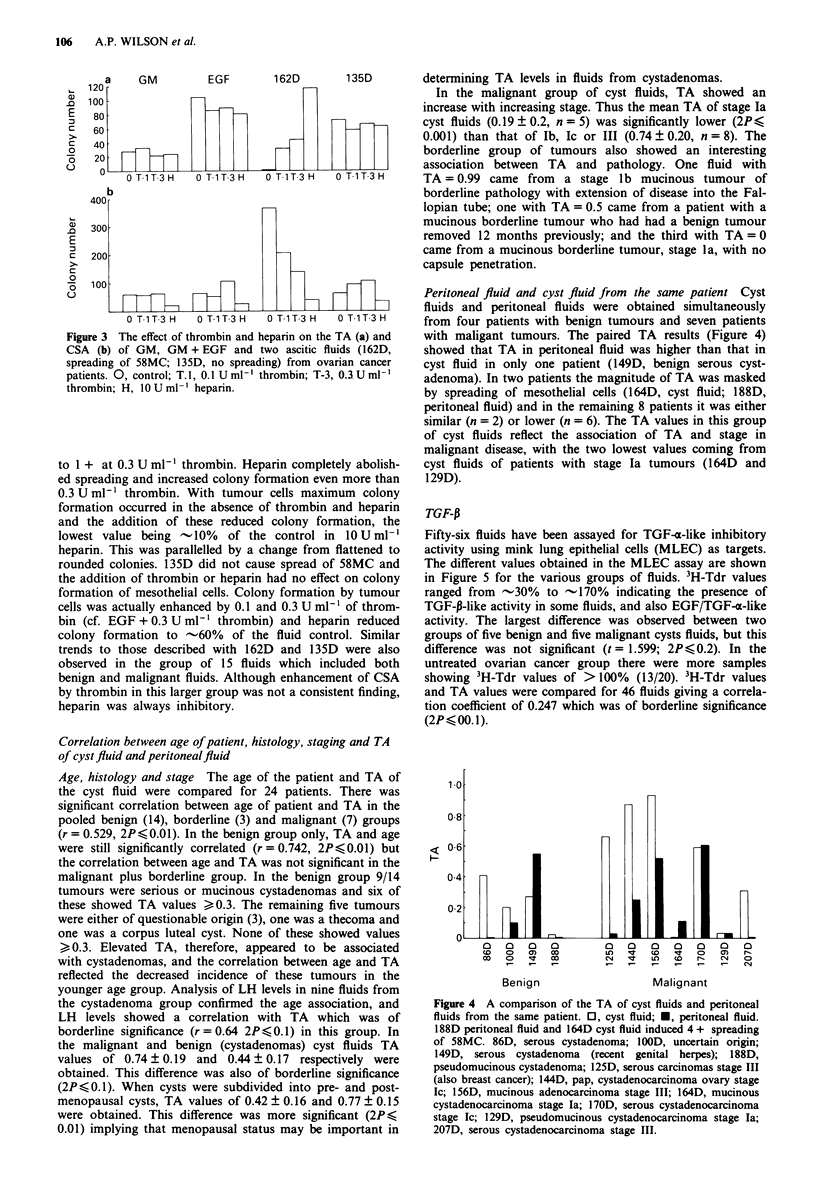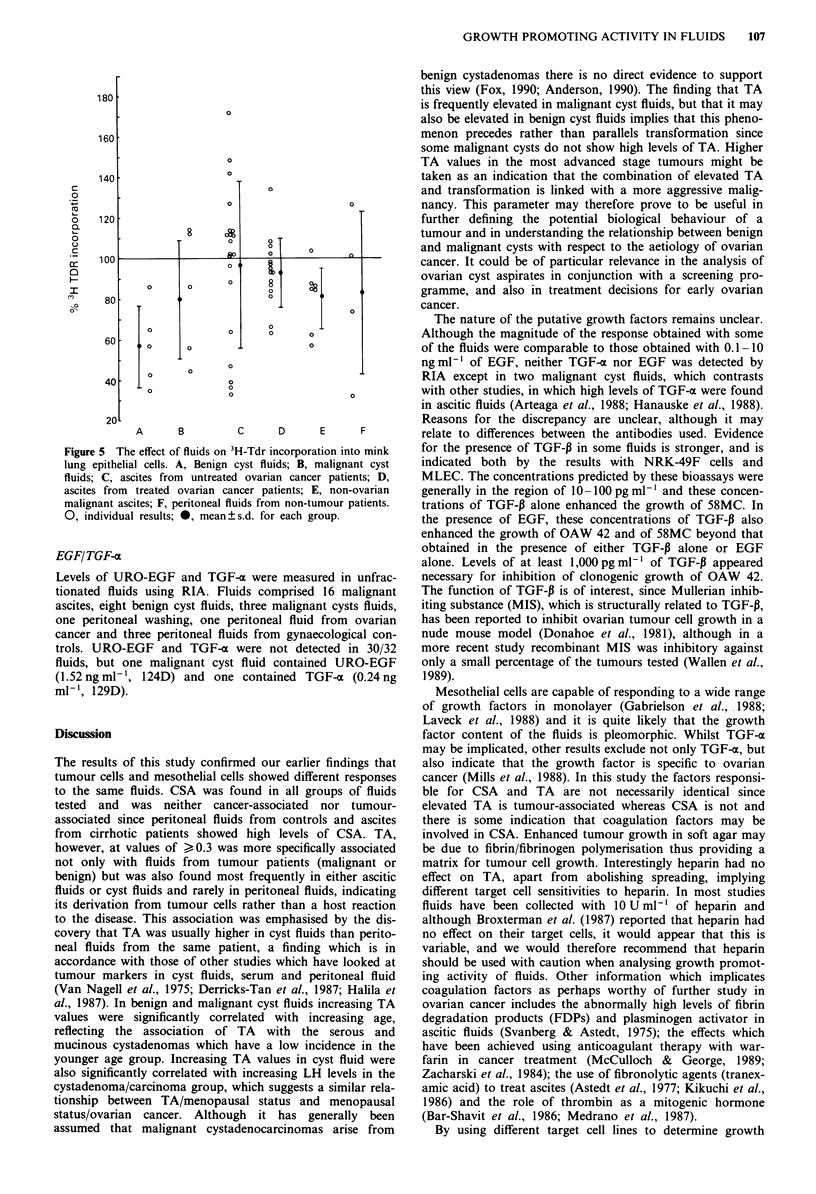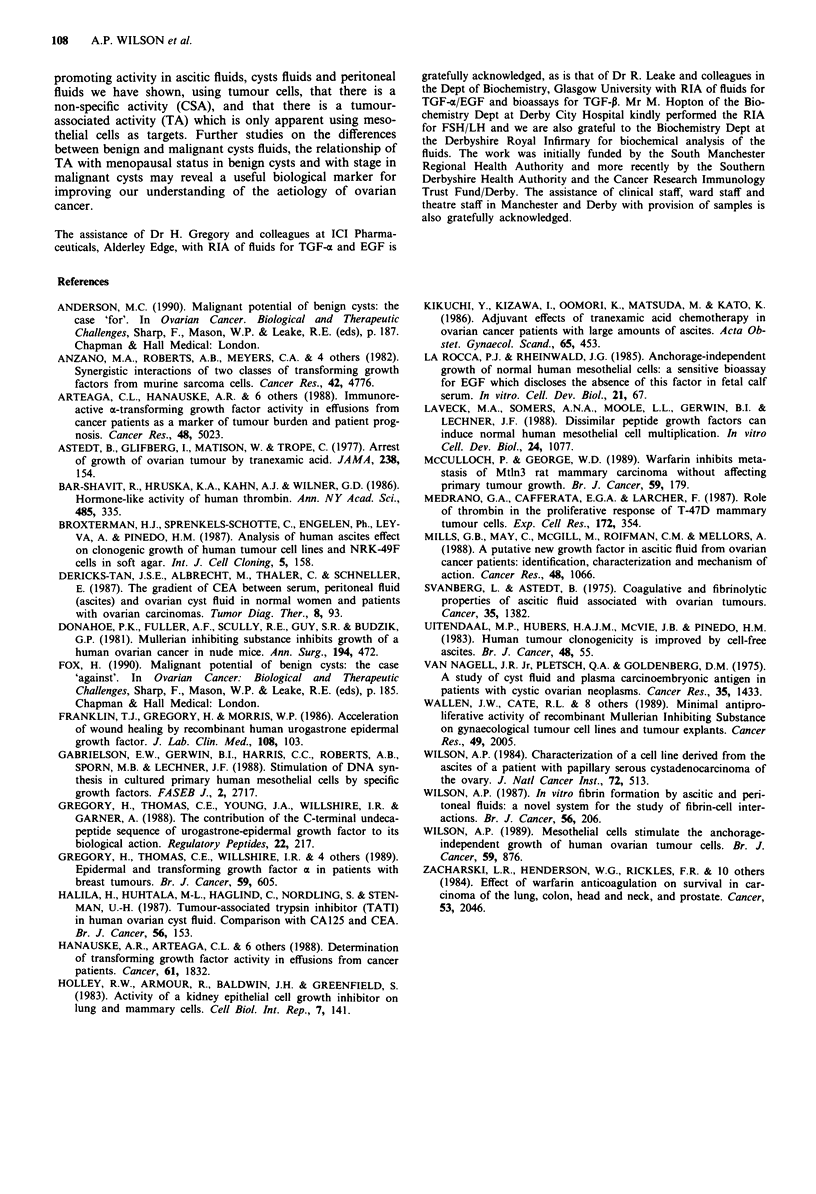# A comparison of the growth promoting properties of ascitic fluids, cyst fluids and peritoneal fluids from patients with ovarian tumours.

**DOI:** 10.1038/bjc.1991.21

**Published:** 1991-01

**Authors:** A. P. Wilson, H. Fox, I. V. Scott, H. Lee, M. Dent, P. R. Golding

**Affiliations:** Oncology Research Laboratory, Derby City Hospital, UK.

## Abstract

**Images:**


Br. J. Cancer (1991), 63, 102-108 © Macmillan Press Ltd., 1991

### A comparison of the growth promoting properties of ascitic fluids, cyst fluids and peritoneal fluids from patients with ovarian tumours

A.P. Wilson[1], H. Fox[2], I.V. Scott[1], H. Lee[1], M. Dent[1] & P.R. Golding[1]

[1]*Oncology Research Laboratory, Derby City Hospital, Uttoxeter Road, Derby DE3 3NE; and* [2]*Department of Reproductive Pathology, St Mary's Hospital, Hathersage Road, Manchester, UK.*

**Summary** The growth promoting properties of ascitic fluids, cyst fluids and peritoneal fluids from patients with ovarian malignancy, benign ovarian tumours and non-tumour related gynaecological conditions have been investigated using an ovarian carcinoma cell line (OAW 42), mesothelial cells (58MC) and rat kidney cells (NRK-49F). Colony stimulating activity (CSA) for tumour cells and transforming activity (TA) for mesothelial cells were weakly correlated, but whereas elevated TA was tumour-associated, CSA was not. However, TA was not cancer-associated and, although the difference between the mean TA values of benign and malignant cyst fluids was of borderline significance, some benign cyst fluids from cystadenomas showed high TA values. Higher levels of TA in the cystadenomas showed a significant correlation with the menopausal status of the patient and higher levels of TA in the malignant cyst fluid/peritoneal fluid groups were associated with more advanced disease. Results indicated that some fluids contained TGF-$\beta$-like activity, but there was no direct evidence for the presence of TGF-$\alpha$/EGF-like activity in the fluids. Heparin inhibited clonogenic growth of tumour cells but not mesothelial cells. The reduced CSA which was observed after treatment of fluids with both heparin and thrombin implicated coagulation factors in the manifestation of CSA. It was concluded that CSA in the fluids was due, at least partly, to fibrin coagulation, and TA was due to unknown growth factor(s) which may include TGF-$\beta$-like activity. The results are discussed in the context of the aetiology of ovarian carcinoma, and the possible clinical significance of TA.

Following reports that cell-free ascites from cancer patients could stimulate tumour cell growth *in vitro* (Uitendaal *et al.*, 1983) there has been considerable interest in the growth promoting properties of these fluids (Broxterman *et al.*, 1987; Arteaga *et al.*, 1988; Hanauske *et al.*, 1988; Mills *et al.*, 1988). In an early study we found that a mesothelial cell line and an ovarian tumour cell line showed different responses to the same fluid. Whereas tumour cells were stimulated by fluids from patients with benign and malignant tumours as well as non-tumour conditions, mesothelial cells were stimulated more specifically by the tumour-related fluids. It therefore appeared that the response of mesothelial cells could be an indicator of the presence of undefined tumour marker(s). The present study was carried out to investigate this possibility using a variety of fluids. These included cyst fluids, ascitic fluids and peritoneal fluids from patients with cancer of the ovary, benign ovarian tumours and gynaecological conditions which were not tumour related. The role of coagulation factors and EGF ± TGF-$\beta$ in the growth promoting effects observed was also investigated particularly with respect to EGF, to which mesothelial cells are known to be sensitive (La Rocca & Rheinwald, 1985).

### Materials and methods

### *Patient groups*

Ascitic fluids were collected from ovarian cancer patients ($n = 42$), non-ovarian cancer patients ($n = 13$), non-tumour patients ($n = 4$) and one patient with a benign ovarian tumour. Peritoneal fluids were collected from gynaecology patients undergoing laporotomy or laporoscopy for non-tumour related conditions ($n = 15$), second-look laporotomies in ovarian cancer patients ($n = 5$), patients with benign ovarian tumours ($n = 8$) and with malignant ovarian tumours ($n = 10$). Cyst fluids were collected from borderline tumours ($n = 3$), benign tumours ($n = 23$) and malignant tumours ($n = 11$). All fluids were collected without heparin, centrifuged at 3,000 r.p.m. for 15 min to remove cells, aliquotted and frozen at −20°C.

Received 27 February 1990; and in revised form 2 July 1990.

### *Target cells*

Growth promoting activity of the fluids was assayed against three target cell populations. These included a mesothelial cell population, 58MC, from the ascites of a patient with ovarian cancer and characterised according to criteria described elsewhere (Wilson, 1989), the normal rat kidney fibroblast cell line, NRK-49F, and one established ovarian tumour cell line, OAW 42 (Wilson, 1984). OAW 42 was maintained as a monolayer culture on growth medium consisting of Dulbecco's Modification of Eagle's medium supplemented with 10% fetal calf serum, 1 mM glutamine, 1 mM sodium pyruvate, 20 IU $l^{-1}$ insulin, penicillin and streptomycin (GM). 58MC was also grown as a monolayer on GM additionally supplemented with 5 ng $ml^{-1}$ epidermal growth factor (EGF) (Gibco) and 0.4 $\mu g\ ml^{-1}$ hydrocortisone (Sigma) (GM + EGF/HC). NRK-49F was maintained as a monolayer on GM without added pyruvate and insulin. All lines were subcultured at weekly intervals and stocks were confirmed to be free of mycoplasma contamination.

### *Soft agar assay*

The growth promoting activity of the fluids was determined under anchorage-independent conditions. One ml base layers of 0.5% agar in GM were prepared in 35 mm dishes (Nunc) and a single cell suspension was overlaid in a 1 ml layer of test fluid containing 0.3% agar. 58MC were added at a final concentration of $10^5$ cells per dish, OAW 42 at $2 \times 10^4$ cells per dish and NRK-49F at $3 \times 10^4$ cells per dish. Controls consisted of the appropriate medium used for routine maintenance of the cell type used. The growth promoting activity of the fluids for tumour cells was designated 'colony stimulating activity' (CSA) and was calculated as:

$$\frac{\text{no. of colonies in test fluid}}{\text{no. of colonies in controls}}$$

Growth promoting activity for the normal cell populations (58MC and NRK-49K) was designated as 'transforming activity' (TA) and was expressed as:

$$\frac{\text{no. of colonies in test fluid}}{\text{no. of colonies in GM + EGF/HC}}$$

Colony formation was expressed as a fraction of colonies formed in the presence of EGF/HC because this gave a standard reference point for defining the mitogenic capability of the normal cell populations, and improved the reproducibility of inter-assay results for TA. Four replicates were routinely included for each test condition and colonies were scored as aggregates of $\geqslant 20$ cells for mesothelial cells, $\geqslant 30$ cells for tumour cells and $\geqslant 50$ cells for NRK-49F cells after 7–10 days incubation at 37°C in an humidified atmosphere containing 5% $CO_2$. Standard deviations were generally $\leqslant 10$–15% of the mean values in each test condition, although higher values did occasionally occur.

*Spreading activity*

Some fluids induced spreading of mesothelial cells in soft agar. Spread cells showed a similar appearance to cells growing in monolayer, exhibiting an elongated shape which was sufficiently flattened to show the nucleus and nucleolus of the cell (Figure 1) (Wilson, 1987). Spreading was scored on a 5-point system as follows: – = no spread, + = a few isolated cells showing spreading, 2 + $\geqslant$10% $\leqslant$50%, 3 + $\geqslant$50% $\leqslant$50%, 4 + $\geqslant$75%. When fluids did cause spreading, colonies were only scored when present as discrete rounded aggregates; colonies were rarely present when fluids caused 4 + spreading.

*Storage time*

Some of the data presented were obtained using ascitic fluid samples which had been stored at –20°C to –40°C for one or more years. Several fluids, assayed at different time points after collection, showed changes in TA. Storage times have therefore been noted for samples within each group to ensure that any differences between groups related to sample differences rather than storage differences.

*Reproducibility*

TA was determined on two separate occasions within 1 month from the period of collection for nine fluids, using 58MC as target cells. The mean values of TA for each group of nine was not significantly different (0.53 ± 0.02 *vs* 0.46 ± 0.16) and the correlation coefficient between paired

**Figure 1** Spreading of 58MC in soft agar (4 + ).

results for each fluid showed a significant relationship ($r = 0.673$; $2P \leqslant 0.01$. $y = 0.504x + 0.189$). Repeat samples obtained from three patients gave similar results (TA = 0.7 and 0.5–5 days between samples; TA = 0.53 and 0.51–14 days between samples; TA = 0.63 and 0.61–1 day between samples).

*Composition of fluids*

The levels of $Na^+$, $K^+$, $Ca^{2+}$, $PO_4^{2-}$, glucose and total protein were determined for 61 fluids on an American Monitor Parallel Analyser. Within each fluid category the different patient groups did not form any distinct subsets (Table I). $Na^+$, $K^+$ and total protein contents were similar in the ascitic fluid, cyst fluid and peritoneal fluid groups. Glucose content was significantly lower in the cyst fluid group, as was $PO_4^{2-}$ content. LH and FSH levels were determined in 65 fluids using a RIA kit (Chelsea Hospital for Women). Results are included only for LH because FSH levels exceeded the range of the assay. The levels of urogastrone (URO-EGF) and TGF-α were determined in 32 unfractionated fluids using methods which have been described elsewhere (Gregory *et al.*, 1988, 1989). Briefly, biosynthetic URO-EGF and TGF-α were derived from synthetic genes expressed in E. coli and purified to give the human sequence 53 and 50 amino acid residues (Frankling *et al.*, 1986; Gregory *et al.*, 1988). RIA assays for URO-EGF and TGF-α were performed using antibodies raised in rabbit and sheep respectively. The standard curves covered the range 20 pg to 10 ng for URO-EGF and 10 pg to 5 ng for TGF-α. Cross-reactivity between URO-EGF and TGF-α was 10 μg = 40 pg for both assays. TGF-β-like activity was determined using a mink lung epithelial cell assay, which shows a dose related inhibition of tritiated thymidine incorporation in response to TGF-β like activity (Holley *et al.*, 1983). In the assay system used 10–1000 pg of TGF-β gave $^3$H-Tdr incorporation values of 80–20% of control values.

*Response of target cells to growth factors*

The responses of 58MC, NRK-49F and OAW 42 to EGF ± TGF-β were determined in a soft agar assay in microplates. Briefly, 100 μl bases of appropriate growth medium (see Target cells) in 0.5% agar were prepared on a 10 x 6 matrix in 96-well microplates. Cells were added as a single cell suspension in a 50 μl overlay in GM containing 0.3% agar, at 0.625 x $10^4$ cells for OAW 42, $10^4$ cells for 58MC and 3 x $10^3$ cells for NRK-49F. Growth factors were added in a 50 μl liquid overlay when the agar was solidified, at 4 x the final required concentration. Final concentrations used were: EGF, 0.1, 1, 10 and 25 ng ml$^{-1}$; TGF-β, 1, 10, 100 and 1,000 pg ml$^{-1}$; and combinations of each concentration of EGF with all concentrations of TGF-β. Replicates of three were used for all test conditions except controls, for which 18 replicates were routinely used. Plates were scored for colonies as described previously.

*Effects of thrombin and heparin on CSA and TA of fluids*

Thrombin (Diagnostic Reagents Ltd) at 0.1 and 0.3 U ml$^{-1}$ was added to 14 fluids. After 3 h incubation at 37°C any clots which had formed were removed and the fluids were then used in the microplate assay described above, using 58MC

**Table I** Summary of biochemical profiles

| | $Na^+$ | $K^+$ | *Glucose* | $Ca^{2+}$ | $PO_4^{2-}$ | *Tot. prot.* |
|---|---|---|---|---|---|---|
| Ascites (39)[a] | 130±12 | 4.1±0.3 | 4.7±1.1 | 1.5±0.4 | 1.1±0.2 | 36±16 |
| Cyst fluids (12) | 141±7 | 4.3±0.4 | 1.5±2.3 | 2.1±0.4 | 0.5±0.5 | 52±19 |
| Peritoneal fluids (10) | 139±3 | 4.4±0.2 | 5.4±0.6 | 2.0±0.1 | 1.1±0.1 | 45±5 |

Plasma ranges are: $Na^+$, 133–145 nmol l$^{-1}$; $K^+$, 3.5–5.0 nmol l$^{-1}$; glucose, 3–6 nmol l$^{-1}$; $Ca^{2+}$, 2.25–2.6 nmol l$^{-1}$; $PO_4^{2-}$, 0.80–1.45 nmol l$^{-1}$; total protein, 59–82 g l$^{-1}$. [a]No. of fluids in group.

and OAW 42 as target cells. Fluids were added as a 150 µl liquid overlay to give a final dilution of 50%. Controls consisted of untreated fluids, GM or GM + EGF/HC with and without added thrombin at 0.1 and 0.3 U $ml^{-1}$. Preservative-free heparin (Monoprin) was also added to fluids at 10 U $ml^{-1}$ and to control media at the same concentrations, and these were included in the same experiments.

### Results

### *Response of target cells to growth factors*

The responses of each of the cell lines are shown in Figure 2a,c,e for EGF and TGF-β added separately and in Figure 2b,d,f for combinations of EGF and TGF-β. Briefly, 58MC and OAW 42 showed an ~4-fold and ~2-fold increase in plating efficiency to 25 ng $ml^{-1}$ of EGF, and NRK-49F also formed colonies (plating efficiency ~2.2%) at this concentration of EGF. Clonogenic growth of 58MC was significantly enhanced by 1, 10 and 100 pg $ml^{-1}$ of TGF-β, whereas OAW 42 was significantly inhibited, (~50%) and only at 1,000 pg $ml^{-1}$. With the combinations, the response of NRK-49F to EGF was significantly enhanced by TGF-β, as expected (Anzano *et al.*, 1982), and TGF-β also enhanced the response of 58MC to 0.1 and 1 ng $ml^{-1}$ of EGF, and of OAW 42 to 1 and 25 ng $ml^{-1}$ of EGF.

### *Transforming activity of fluids for mesothelial cells*

A total of 135 fluids have been tested at 50% dilution for transforming activity (TA) in a soft agar assay against one mesothelial cell population (58MC). Results are shown in Table II for the different patient groups which were investigated.

Mean TA values in the different tumour ascites groups were significantly higher than the mean TA value for the control GM group, but the mean TA of the non-tumour group (liver cirrhosis) was not significantly different from the control, indicating that higher levels of TA are tumour-associated. There were no significant differences between TA values in the different ascites sub-groups. In all the peritoneal fluid groups, mean TA values were not significantly different from the control value, although within each sub-group there was a small percentage of fluids that did have higher TA values. Most notable was a peritoneal fluid sample (volume 1.5 ml) from a 2LL-patient with residual disease (TA = 1.32). In the cyst fluid group the difference between benign and malignant cyst fluids reached borderline significance (storage times for the two groups were not significantly different, see Materials and methods), with some benign cyst fluids having high TA values and some malignant cyst fluids having low TA values. Two borderline cysts also showed higher TA values. Because of the difference between benign and malig-

**Figure 2** The response of target cell lines to EGF and TGF-β. **a,b** = 58MC; **c,d** = OAW 42; **e,f** = NRK-49F. The horizontal axis (log scale) for graphs a, c, e shows EGF at 0.1, 1, 10 and 25 ng $ml^{-1}$ (●) and TGF-β at 1, 10, 100 and 1,000 pg $ml^{-1}$ (○). PE% = percentage plating efficiency. **a,c,e** = EGF ● TGF-β ○. **b,d,f** = combinations of TGF-β (horizontal axis) and EGF: ○, 25 ng $ml^{-1}$; ■, 10 ng $ml^{-1}$; □, 1 ng $ml^{-1}$; ●, 0.1 ng $ml^{-1}$. The horizontal dashed line on each graph shows the plating efficiency of target cells in the presence of growth medium (CSA = 1; **c, d**) or in the presence of 2.5 ng $ml^{-1}$ of EGF (TA = 1; **a,b/e,f**).

nant cyst fluids, the number of fluids showing values ⩾0.3 was determined in each group. There was only 1/15 in the peritoneal fluid control group contrasting with 10/13 in the untreated ovarian cancer ascites group. The number was also slightly higher in the malignant cyst fluid group (7/11, 64%) than in the benign cyst fluid group (11/23, 48%).

*Colony stimulating activity (CSA) of fluids of ovarian tumour cells*

A total of 58 fluids have been tested at 50% dilution for CSA in a soft agar assay, using OAW 42 as target cells (Table II). CSA was generally higher in the different tumour ascites groups (range 0–24), but the ability to promote tumour cell growth was not restricted to a clinical tumour condition since some peritoneal fluids from the gynaecological group also showed this ability (range 0–21), and the highest values were present in ascites from patients with liver failure (range 2.9–72). There was, however, significant correlation between CSA and TA ($r=0.345$, $n=44$; $2P \leqslant 0.05$).

*Transforming activity of fluids for NRK-49F cells*

A total of 18 fluids were assayed at 50% dilution simultaneously against NRK-49F and 58MC for TA. Seven fluids induced 4+ spreading of cells in soft agar (see Materials and methods) and were excluded from the analysis. In the remaining 11 pairs of results there was no significant correlation ($r=0.135$). In 4/6 ascitic fluids and 1/3 malignant cyst fluids there were large differences between TA values obtained with the two lines. In 4/5 cases (three ascites and one cyst fluid) this showed as a high value for NRK-49F (2–5) and a low value for 58MC (0.014–0.51), and in one case (ascites) a high value for 58MC (1.41) and a low value for NRK-49F (0.62).

*Correlation between induction of spreading of mesothelial cells in soft agar and CSA of fluids for OAW 42*

A number of fluids induced spreading of mesothelial cells in soft agar (see Materials and methods) and this was more frequent in certain fluid groups. For final comparison between fluids results were separated into two categories: −/+ and 2+/4+. The highest incidence of 2+/4+ spreading occurred in the control group of peritoneal fluids (15/21, 71%), ascites from the treated group of ovarian cancer patients (9/14, 64%) and peritoneal fluids from 2LL patients (3/7, 42%). The lowest incidence of spreading occurred in the cyst fluids (1/31, 3%). Although fluids which induced spreading of 58MC in soft agar did not induce a monolayer-like appearance of tumour cells, some flattening in colony morphology of OAW 42 was observed and it was further noted that the CSA of fluids which induced 2+/4+ spreading of mesothelial cells was significantly higher than that of fluids which did not (see Table III). The exceptions were the four fluids from patients with liver failure which showed high levels of CSA but did not cause spreading of mesothelial cells.

*Effect of thrombin and heparin on CSA and TA of fluids*

Because the spread of 58MC in soft agar was believed to be due to coagulation of fibrinogen with subsequent attachment by mesothelial cells to the fibrin, the effects of preventing fibrin formation by the addition of 0.1 and 0.3 U ml$^{-1}$ of thrombin or 10 U ml$^{-1}$ of preservative-free heparin were tested on the CSA and TA of 15 fluids. Results are shown in Figure 3 for the response of mesothelial cells (a) and tumour cells (b) to GM, GM + EGF and two representative fluids, one of which caused spreading of 58MC (162D) and one of which did not (135D).

Thrombin and heparin had no significant effect on the colony formation of mesothelial cells in the presence or absence of 2.5 ng ml$^{-1}$ of EGF, to which the mesothelial cells showed a 3.8-fold increase in colony formation. Heparin, at 10 U ml$^{-1}$, significantly reduced the colony formation of tumour cells both in the presence and absence of 2.5 ng ml$^{-1}$ of EGF. The tumour cells did not show any response to this concentration of EGF, but a 2-fold increase in colony formation was observed in the presence of EGF and 0.3 U ml$^{-1}$ of thrombin. With 162D, the addition of thrombin increased the colony formation of 58MC and reduced spreading from 4+

**Table III** A comparison of CSA for OAW 42 and spreading activity for 58MC

| Exp. No. | CSA: No spread − | CSA: Spreading +/4+ | $t$ | 2P |
|---|---|---|---|---|
| 1. | 1.5±1.03 (16)[a] | 5.6±7.7 (11) | −2.121 | ⩽0.05 |
| 2. | 2.9±1.90 (11) | 6.6±1.1 (2) | −2.614 | ⩽0.02 |
| 3. | 1.2±0.10 (3) | 2.6±1.1 (8) | −2.129 | ⩽0.05 |

Results (mean±s.d.) are shown for three separate experiments in which a number of fluids were tested simultaneously against OAW 42 and 58MC. ( ),[a] no. fluids in group.

**Table II** Transforming activity (TA) of peritoneal fluids, cyst fluids and effusions for mesothelial cells and colony stimulating activity (CSA) for ovarian cancer cells (OAW 42)

| Source | TA | Range | >0.3 | CSA | Range |
|---|---|---|---|---|---|
| *Peritoneal fluids* | | | | | |
| Gyn. patients | 0.15±0.12 (15) | 0–0.47 | 1 (7%) | 3.1–1.1 (4) | 1.8–4.2 |
| Benign ovarian | 0.25±0.28 (8) | 0–0.80 | 3 (37%) | 21 (1) | 21 |
| Ovarian cancer | 0.18±0.20 (10) | 0–0.54 | 2 (20%) | 0, 1.1 (2) | 0–1.1 |
| 2-LL | 0.35±0.55 (5) | 0.03–1.32 | 1 (20%) | 2.5 (1) | 2.5 |
| *Cyst fluids* | | | | | |
| Benign ovarian | 0.29±0.17 (23)[a] | 0–0.70 | 11 (48%) | 1.4–1.1 (8) | 0–3.1 |
| Borderline | 0.50±0.50 (3)[a] | 0–0.99 | 2 (67%) | 0, 2 (2) | 0–2 |
| Ovarian cancer | 0.46±0.38 (11)[b] | 0–0.93 | 7 (64%) | 1.3±0.6 (5) | 0.9–2.3 |
| *Ascites* | | | | | |
| Liver cirrhosis | 0.18±0.14 (4) | 0–0.35 | 1 (25%) | 18.4±30 (5) | 2.9–72 |
| Benign ovarian | 0.66 (1) | 0.66 | 1 (100%) | | |
| Ovarian cancer, untreated | 0.48±0.23 (13)[d] | 0.18–0.98 | 10 (77%) | 3.1±4.0 (8) | 0–12.5 |
| Ovarian cancer, treated | 0.44±0.28 (29)[c] | 0–1.22 | 21 (72%) | 3.7±4.7 (17) | 0–21 |
| Non-ovarian cancer | 0.74±1.00 (13)[a] | 0–3.80 | 9 (69%) | 4.0±2.0 (9) | 0.7–6.4 |

( ) no. of fluids tested in group for TA or CSA. Significance limits: $2P \leqslant$ [a]0.10; [b]0.025; [c]0.005; [d]0.001. Mean TA for GM (colonies in GM/colonies in EGF/HC) was 0.16±0.14 ($n=11$).

**Figure 3** The effect of thrombin and heparin on the TA (**a**) and CSA (**b**) of GM, GM + EGF and two ascitic fluids (162D, spreading of 58MC; 135D, no spreading) from ovarian cancer patients. O, control; T.1, 0.1 U $ml^{-1}$ thrombin; T-3, 0.3 U $ml^{-1}$ thrombin; H, 10 U $ml^{-1}$ heparin.

to 1 + at 0.3 U $ml^{-1}$ thrombin. Heparin completely abolished spreading and increased colony formation even more than 0.3 U $ml^{-1}$ thrombin. With tumour cells maximum colony formation occurred in the absence of thrombin and heparin and the addition of these reduced colony formation, the lowest value being ~10% of the control in 10 U $ml^{-1}$ heparin. This was parallelled by a change from flattened to rounded colonies. 135D did not cause spread of 58MC and the addition of thrombin or heparin had no effect on colony formation of mesothelial cells. Colony formation by tumour cells was actually enhanced by 0.1 and 0.3 U $ml^{-1}$ of thrombin (cf. EGF + 0.3 U $ml^{-1}$ thrombin) and heparin reduced colony formation to ~60% of the fluid control. Similar trends to those described with 162D and 135D were also observed in the group of 15 fluids which included both benign and malignant fluids. Although enhancement of CSA by thrombin in this larger group was not a consistent finding, heparin was always inhibitory.

### *Correlation between age of patient, histology, staging and TA of cyst fluid and peritoneal fluid*

*Age, histology and stage* The age of the patient and TA of the cyst fluid were compared for 24 patients. There was significant correlation between age of patient and TA in the pooled benign (14), borderline (3) and malignant (7) groups ($r = 0.529$, $2P \leqslant 0.01$). In the benign group only, TA and age were still significantly correlated ($r = 0.742$, $2P \leqslant 0.01$) but the correlation between age and TA was not significant in the malignant plus borderline group. In the benign group 9/14 tumours were serious or mucinous cystadenomas and six of these showed TA values $\geqslant 0.3$. The remaining five tumours were either of questionable origin (3), one was a thecoma and one was a corpus luteal cyst. None of these showed values $\geqslant 0.3$. Elevated TA, therefore, appeared to be associated with cystadenomas, and the correlation between age and TA reflected the decreased incidence of these tumours in the younger age group. Analysis of LH levels in nine fluids from the cystadenoma group confirmed the age association, and LH levels showed a correlation with TA which was of borderline significance ($r = 0.64$ $2P \leqslant 0.1$) in this group. In the malignant and benign (cystadenomas) cyst fluids TA values of $0.74 \pm 0.19$ and $0.44 \pm 0.17$ respectively were obtained. This difference was also of borderline significance ($2P \leqslant 0.1$). When cysts were subdivided into pre- and postmenopausal cysts, TA values of $0.42 \pm 0.16$ and $0.77 \pm 0.15$ were obtained. This difference was more significant ($2P \leqslant 0.01$) implying that menopausal status may be important in determining TA levels in fluids from cystadenomas.

In the malignant group of cyst fluids, TA showed an increase with increasing stage. Thus the mean TA of stage Ia cyst fluids ($0.19 \pm 0.2$, $n = 5$) was significantly lower ($2P \leqslant 0.001$) than that of Ib, Ic or III ($0.74 \pm 0.20$, $n = 8$). The borderline group of tumours also showed an interesting association between TA and pathology. One fluid with TA = 0.99 came from a stage 1b mucinous tumour of borderline pathology with extension of disease into the Fallopian tube; one with TA = 0.5 came from a patient with a mucinous borderline tumour who had had a benign tumour removed 12 months previously; and the third with TA = 0 came from a mucinous borderline tumour, stage 1a, with no capsule penetration.

*Peritoneal fluid and cyst fluid from the same patient* Cyst fluids and peritoneal fluids were obtained simultaneously from four patients with benign tumours and seven patients with maligant tumours. The paired TA results (Figure 4) showed that TA in peritoneal fluid was higher than that in cyst fluid in only one patient (149D, benign serous cystadenoma). In two patients the magnitude of TA was masked by spreading of mesothelial cells (164D, cyst fluid; 188D, peritoneal fluid) and in the remaining 8 patients it was either similar ($n = 2$) or lower ($n = 6$). The TA values in this group of cyst fluids reflect the association of TA and stage in malignant disease, with the two lowest values coming from cyst fluids of patients with stage Ia tumours (164D and 129D).

### *TGF-β*

Fifty-six fluids have been assayed for TGF-α-like inhibitory activity using mink lung epithelial cells (MLEC) as targets. The different values obtained in the MLEC assay are shown in Figure 5 for the various groups of fluids. $^3$H-Tdr values ranged from ~30% to ~170% indicating the presence of TGF-β-like activity in some fluids, and also EGF/TGF-α-like activity. The largest difference was observed between two groups of five benign and five malignant cysts fluids, but this difference was not significant ($t = 1.599$; $2P \leqslant 0.2$). In the untreated ovarian cancer group there were more samples showing $^3$H-Tdr values of $> 100\%$ (13/20). $^3$H-Tdr values and TA values were compared for 46 fluids giving a correlation coefficient of 0.247 which was of borderline significance ($2P \leqslant 00.1$).

**Figure 4** A comparison of the TA of cyst fluids and peritoneal fluids from the same patient. □, cyst fluid; ■, peritoneal fluid. 188D peritoneal fluid and 164D cyst fluid induced 4 + spreading of 58MC. 86D, serous cystadenoma; 100D, uncertain origin; 149D, serous cystadenoma (recent genital herpes); 188D, pseudomucinous cystadenoma; 125D, serous carcinomas stage III (also breast cancer); 144D, pap, cystadenocarcinoma ovary stage Ic; 156D, mucinous adenocarcinoma stage III; 164D, mucinous cystadenocarcinoma stage Ia; 170D, serous cystadenocarcinoma stage Ic; 129D, pseudomucinous cystadenocarcinoma stage Ia; 207D, serous cystadenocarcinoma stage III.

**Figure 5** The effect of fluids on $^{3}$H-Tdr incorporation into mink lung epithelial cells. **A**, Benign cyst fluids; **B**, malignant cyst fluids; **C**, ascites from untreated ovarian cancer patients; **D**, ascites from treated ovarian cancer patients; **E**, non-ovarian malignant ascites; **F**, peritoneal fluids from non-tumour patients. O, individual results; ●, mean ± s.d. for each group.

*EGF/TGF-α*

Levels of URO-EGF and TGF-α were measured in unfractionated fluids using RIA. Fluids comprised 16 malignant ascites, eight benign cyst fluids, three malignant cysts fluids, one peritoneal washing, one peritoneal fluid from ovarian cancer and three peritoneal fluids from gynaecological controls. URO-EGF and TGF-α were not detected in 30/32 fluids, but one malignant cyst fluid contained URO-EGF (1.52 ng ml$^{-1}$, 124D) and one contained TGF-α (0.24 ng ml$^{-1}$, 129D).

### Discussion

The results of this study confirmed our earlier findings that tumour cells and mesothelial cells showed different responses to the same fluids. CSA was found in all groups of fluids tested and was neither cancer-associated nor tumour-associated since peritoneal fluids from controls and ascites from cirrhotic patients showed high levels of CSA. TA, however, at values of $\geqslant 0.3$ was more specifically associated not only with fluids from tumour patients (malignant or benign) but was also found most frequently in either ascitic fluids or cyst fluids and rarely in peritoneal fluids, indicating its derivation from tumour cells rather than a host reaction to the disease. This association was emphasised by the discovery that TA was usually higher in cyst fluids than peritoneal fluids from the same patient, a finding which is in accordance with those of other studies which have looked at tumour markers in cyst fluids, serum and peritoneal fluid (Van Nagell *et al.*, 1975; Derricks-Tan *et al.*, 1987; Halila *et al.*, 1987). In benign and malignant cyst fluids increasing TA values were significantly correlated with increasing age, reflecting the association of TA with the serous and mucinous cystadenomas which have a low incidence in the younger age group. Increasing TA values in cyst fluid were also significantly correlated with increasing LH levels in the cystadenoma/carcinoma group, which suggests a similar relationship between TA/menopausal status and menopausal status/ovarian cancer. Although it has generally been assumed that malignant cystadenocarcinomas arise from benign cystadenomas there is no direct evidence to support this view (Fox, 1990; Anderson, 1990). The finding that TA is frequently elevated in malignant cyst fluids, but that it may also be elevated in benign cyst fluids implies that this phenomenon precedes rather than parallels transformation since some malignant cysts do not show high levels of TA. Higher TA values in the most advanced stage tumours might be taken as an indication that the combination of elevated TA and transformation is linked with a more aggressive malignancy. This parameter may therefore prove to be useful in further defining the potential biological behaviour of a tumour and in understanding the relationship between benign and malignant cysts with respect to the aetiology of ovarian cancer. It could be of particular relevance in the analysis of ovarian cyst aspirates in conjunction with a screening programme, and also in treatment decisions for early ovarian cancer.

The nature of the putative growth factors remains unclear. Although the magnitude of the response obtained with some of the fluids were comparable to those obtained with 0.1–10 ng ml$^{-1}$ of EGF, neither TGF-α nor EGF was detected by RIA except in two malignant cyst fluids, which contrasts with other studies, in which high levels of TGF-α were found in ascitic fluids (Arteaga *et al.*, 1988; Hanauske *et al.*, 1988). Reasons for the discrepancy are unclear, although it may relate to differences between the antibodies used. Evidence for the presence of TGF-β in some fluids is stronger, and is indicated both by the results with NRK-49F cells and MLEC. The concentrations predicted by these bioassays were generally in the region of 10–100 pg ml$^{-1}$ and these concentrations of TGF-β alone enhanced the growth of 58MC. In the presence of EGF, these concentrations of TGF-β also enhanced the growth of OAW 42 and of 58MC beyond that obtained in the presence of either TGF-β alone or EGF alone. Levels of at least 1,000 pg ml$^{-1}$ of TGF-β appeared necessary for inhibition of clonogenic growth of OAW 42. The function of TGF-β is of interest, since Mullerian inhibiting substance (MIS), which is structurally related to TGF-β, has been reported to inhibit ovarian tumour cell growth in a nude mouse model (Donahoe *et al.*, 1981), although in a more recent study recombinant MIS was inhibitory against only a small percentage of the tumours tested (Wallen *et al.*, 1989).

Mesothelial cells are capable of responding to a wide range of growth factors in monolayer (Gabrielson *et al.*, 1988; Laveck *et al.*, 1988) and it is quite likely that the growth factor content of the fluids is pleomorphic. Whilst TGF-α may be implicated, other results exclude not only TGF-α, but also indicate that the growth factor is specific to ovarian cancer (Mills *et al.*, 1988). In this study the factors responsible for CSA and TA are not necessarily identical since elevated TA is tumour-associated whereas CSA is not and there is some indication that coagulation factors may be involved in CSA. Enhanced tumour growth in soft agar may be due to fibrin/fibrinogen polymerisation thus providing a matrix for tumour cell growth. Interestingly heparin had no effect on TA, apart from abolishing spreading, implying different target cell sensitivities to heparin. In most studies fluids have been collected with 10 U ml$^{-1}$ of heparin and although Broxterman *et al.* (1987) reported that heparin had no effect on their target cells, it would appear that this is variable, and we would therefore recommend that heparin should be used with caution when analysing growth promoting activity of fluids. Other information which implicates coagulation factors as perhaps worthy of further study in ovarian cancer includes the abnormally high levels of fibrin degradation products (FDPs) and plasminogen activator in ascitic fluids (Svanberg & Astedt, 1975); the effects which have been achieved using anticoagulant therapy with warfarin in cancer treatment (McCulloch & George, 1989; Zacharski *et al.*, 1984); the use of fibronolytic agents (tranexamic acid) to treat ascites (Astedt *et al.*, 1977; Kikuchi *et al.*, 1986) and the role of thrombin as a mitogenic hormone (Bar-Shavit *et al.*, 1986; Medrano *et al.*, 1987).

By using different target cell lines to determine growth promoting activity in ascitic fluids, cysts fluids and peritoneal fluids we have shown, using tumour cells, that there is a non-specific activity (CSA), and that there is a tumour-associated activity (TA) which is only apparent using mesothelial cells as targets. Further studies on the differences between benign and malignant cysts fluids, the relationship of TA with menopausal status in benign cysts and with stage in malignant cysts may reveal a useful biological marker for improving our understanding of the aetiology of ovarian cancer.

The assistance of Dr H. Gregory and colleagues at ICI Pharmaceuticals, Alderley Edge, with RIA of fluids for TGF-α and EGF is gratefully acknowledged, as is that of Dr R. Leake and colleagues in the Dept of Biochemistry, Glasgow University with RIA of fluids for TGF-α/EGF and bioassays for TGF-β. Mr M. Hopton of the Biochemistry Dept at Derby City Hospital kindly performed the RIA for FSH/LH and we are also grateful to the Biochemistry Dept at the Derbyshire Royal Infirmary for biochemical analysis of the fluids. The work was initially funded by the South Manchester Regional Health Authority and more recently by the Southern Derbyshire Health Authority and the Cancer Research Immunology Trust Fund/Derby. The assistance of clinical staff, ward staff and theatre staff in Manchester and Derby with provision of samples is also gratefully acknowledged.

### References

ANDERSON, M.C. (1990). Malignant potential of benign cysts: the case 'for'. In *Ovarian Cancer. Biological and Therapeutic Challenges*, Sharp, F., Mason, W.P. & Leake, R.E. (eds), p. 187. Chapman & Hall Medical: London.

ANZANO, M.A., ROBERTS, A.B., MEYERS, C.A. & 4 others (1982). Synergistic interactions of two classes of transforming growth factors from murine sarcoma cells. *Cancer Res.*, **42**, 4776.

ARTEAGA, C.L., HANAUSKE, A.R. & 6 others (1988). Immunoreactive α-transforming growth factor activity in effusions from cancer patients as a marker of tumour burden and patient prognosis. *Cancer Res.*, **48**, 5023.

ASTEDT, B., GLIFBERG, I., MATISON, W. & TROPE, C. (1977). Arrest of growth of ovarian tumour by tranexamic acid. *JAMA*, **238**, 154.

BAR-SHAVIT, R., HRUSKA, K.A., KAHN, A.J. & WILNER, G.D. (1986). Hormone-like activity of human thrombin. *Ann. NY Acad. Sci.*, **485**, 335.

BROXTERMAN, H.J., SPRENKELS-SCHOTTE, C., ENGELEN, Ph., LEYVA, A. & PINEDO, H.M. (1987). Analysis of human ascites effect on clonogenic growth of human tumour cell lines and NRK-49F cells in soft agar. *Int. J. Cell Cloning*, **5**, 158.

DERICKS-TAN, J.S.E., ALBRECHT, M., THALER, C. & SCHNELLER, E. (1987). The gradient of CEA between serum, peritoneal fluid (ascites) and ovarian cyst fluid in normal women and patients with ovarian carcinomas. *Tumor Diag. Ther.*, **8**, 93.

DONAHOE, P.K., FULLER, A.F., SCULLY, R.E., GUY, S.R. & BUDZIK, G.P. (1981). Mullerian inhibiting substance inhibits growth of a human ovarian cancer in nude mice. *Ann. Surg.*, **194**, 472.

FOX, H. (1990). Malignant potential of benign cysts: the case 'against'. In *Ovarian Cancer: Biological and Therapeutic Challenges*, Sharp, F., Mason, W.P. & Leake, R.E. (eds), p. 185. Chapman & Hall Medical: London.

FRANKLIN, T.J., GREGORY, H. & MORRIS, W.P. (1986). Acceleration of wound healing by recombinant human urogastrone epidermal growth factor. *J. Lab. Clin. Med.*, **108**, 103.

GABRIELSON, E.W., GERWIN, B.I., HARRIS, C.C., ROBERTS, A.B., SPORN, M.B. & LECHNER, J.F. (1988). Stimulation of DNA synthesis in cultured primary human mesothelial cells by specific growth factors. *FASEB J.*, **2**, 2717.

GREGORY, H., THOMAS, C.E., YOUNG, J.A., WILLSHIRE, I.R. & GARNER, A. (1988). The contribution of the C-terminal undecapeptide sequence of urogastrone-epidermal growth factor to its biological action. *Regulatory Peptides*, **22**, 217.

GREGORY, H., THOMAS, C.E., WILLSHIRE, I.R. & 4 others (1989). Epidermal and transforming growth factor α in patients with breast tumours. *Br. J. Cancer*, **59**, 605.

HALILA, H., HUHTALA, M-L., HAGLIND, C., NORDLING, S. & STENMAN, U.-H. (1987). Tumour-associated trypsin inhibitor (TATI) in human ovarian cyst fluid. Comparison with CA125 and CEA. *Br. J. Cancer*, **56**, 153.

HANAUSKE, A.R., ARTEAGA, C.L. & 6 others (1988). Determination of transforming growth factor activity in effusions from cancer patients. *Cancer*, **61**, 1832.

HOLLEY, R.W., ARMOUR, R., BALDWIN, J.H. & GREENFIELD, S. (1983). Activity of a kidney epithelial cell growth inhibitor on lung and mammary cells. *Cell Biol. Int. Rep.*, **7**, 141.

KIKUCHI, Y., KIZAWA, I., OOMORI, K., MATSUDA, M. & KATO, K. (1986). Adjuvant effects of tranexamic acid chemotherapy in ovarian cancer patients with large amounts of ascites. *Acta Obstet. Gynaecol. Scand.*, **65**, 453.

LA ROCCA, P.J. & RHEINWALD, J.G. (1985). Anchorage-independent growth of normal human mesothelial cells: a sensitive bioassay for EGF which discloses the absence of this factor in fetal calf serum. *In vitro. Cell. Dev. Biol.*, **21**, 67.

LAVECK, M.A., SOMERS, A.N.A., MOOLE, L.L., GERWIN, B.I. & LECHNER, J.F. (1988). Dissimilar peptide growth factors can induce normal human mesothelial cell multiplication. *In vitro Cell. Dev. Biol.*, **24**, 1077.

MCCULLOCH, P. & GEORGE, W.D. (1989). Warfarin inhibits metastasis of Mtln3 rat mammary carcinoma without affecting primary tumour growth. *Br. J. Cancer*, **59**, 179.

MEDRANO, G.A., CAFFERATA, E.G.A. & LARCHER, F. (1987). Role of thrombin in the proliferative response of T-47D mammary tumour cells. *Exp. Cell Res.*, **172**, 354.

MILLS, G.B., MAY, C., MCGILL, M., ROIFMAN, C.M. & MELLORS, A. (1988). A putative new growth factor in ascitic fluid from ovarian cancer patients: identification, characterization and mechanism of action. *Cancer Res.*, **48**, 1066.

SVANBERG, L. & ASTEDT, B. (1975). Coagulative and fibrinolytic properties of ascitic fluid associated with ovarian tumours. *Cancer*, **35**, 1382.

UITENDAAL, M.P., HUBERS, H.A.J.M., MCVIE, J.B. & PINEDO, H.M. (1983). Human tumour clonogenicity is improved by cell-free ascites. *Br. J. Cancer*, **48**, 55.

VAN NAGELL, J.R. Jr, PLETSCH, Q.A. & GOLDENBERG, D.M. (1975). A study of cyst fluid and plasma carcinoembryonic antigen in patients with cystic ovarian neoplasms. *Cancer Res.*, **35**, 1433.

WALLEN, J.W., CATE, R.L. & 8 others (1989). Minimal antiproliferative activity of recombinant Mullerian Inhibiting Substance on gynaecological tumour cell lines and tumour explants. *Cancer Res.*, **49**, 2005.

WILSON, A.P. (1984). Characterization of a cell line derived from the ascites of a patient with papillary serous cystadenocarcinoma of the ovary. *J. Natl Cancer Inst.*, **72**, 513.

WILSON, A.P. (1987). *In vitro* fibrin formation by ascitic and peritoneal fluids: a novel system for the study of fibrin-cell interactions. *Br. J. Cancer*, **56**, 206.

WILSON, A.P. (1989). Mesothelial cells stimulate the anchorage-independent growth of human ovarian tumour cells. *Br. J. Cancer*, **59**, 876.

ZACHARSKI, L.R., HENDERSON, W.G., RICKLES, F.R. & 10 others (1984). Effect of warfarin anticoagulation on survival in carcinoma of the lung, colon, head and neck, and prostate. *Cancer*, **53**, 2046.